# Localization of Hemostasis Elements in Aspirated Coronary Thrombi at Different Stages of Evolution

**DOI:** 10.3390/ijms252111746

**Published:** 2024-11-01

**Authors:** Dóra Pituk, László Balogh, Emőke Horváth, Zoltán Hegyi, Barbara Baráth, Réka Bogáti, Péter Szűcs, Zoltán Papp, Éva Katona, Zsuzsanna Bereczky

**Affiliations:** 1 Division of Clinical Laboratory Science, Department of Laboratory Medicine, Faculty of Medicine, University of Debrecen, 4032 Debrecen, Hungary; pituk.dora@med.unideb.hu (D.P.); barath.barbi11@gmail.com (B.B.); bogati.reka@med.unideb.hu (R.B.); ekatona@med.unideb.hu (É.K.); 2 Kálmán Laki Doctoral School, Faculty of Medicine, University of Debrecen, 4032 Debrecen, Hungary; 3 Department of Cardiology, Faculty of Medicine, University of Debrecen, 4032 Debrecen, Hungary; laszlobalogh76@yahoo.com (L.B.); pappz@med.unideb.hu (Z.P.); 4 Department of Pathology, Faculty of Medicine, George Emil Palade University of Medicine, Pharmacy, Science, and Technology of Targu Mures, 540142 Targu Mures, Romania; horvath_emoke@yahoo.com; 5 Department of Anatomy, Histology and Embryology, Faculty of Medicine, University of Debrecen, 4032 Debrecen, Hungary; hegyiz@anat.med.unideb.hu (Z.H.); szucs.peter@med.unideb.hu (P.S.)

**Keywords:** thrombus aspiration, coronary thrombosis, NETs (neutrophil extracellular traps), histopathology, myocardial Infarction, protein C, factor XIII, α2-plasmin inhibitor, fibrinogen, confocal microscopy

## Abstract

The structure of aspirated coronary thrombus in ST-segment elevation myocardial infarction (STEMI) is still being studied. Our aims were to characterize coronary thrombi of different ages, focusing on the appearance of activated protein C (APC/PC) and its relation to the elements of neutrophil extracellular traps (NETs), and the factors closely related to fibrin as factor XIII (FXIII) and α2 plasmin inhibitor (α2-PI). The thrombi of *n* = 24 male patients with atherosclerotic coronary plaque rupture related to native coronary artery occlusion were selected for histopathology analysis. Thrombus age was distinguished as fresh, lytic, and organized, and then analyzed by immunofluorescent staining and confocal microscopy. FXIII was present at a high level and showed a high degree of co-localization with fibrin in all stages of thrombus evolution. The amount of α2-PI was low in the fresh thrombi, which increased significantly to the lytic phase. It was evenly distributed and consistently associated with fibrin. APC/PC appeared in the fresh thrombus and remained constant during its evolution. The presence of NET marker and CD66b was most dominant in the lytic phase. APC/PC co-localization with the elements of NET formation shows its role in NET degradation. These observations suggest the importance of searching for further targeted therapeutic strategies in STEMI patients.

## 1. Introduction

Plaque disruption and coronary thrombus formation are the main mechanisms of acute coronary syndrome. In ST-elevation myocardial infarction (STEMI), manual thrombectomy and thrombus aspiration used to be recommended with class IIa indication according to the 2008 ESC guideline (TAPAS study) [1]; however, it was downgraded in 2014 to class IIb indication (TASTE study) [2] and it is not routinely indicated (class of recommendation is IIIa) since 2018 based on the results of the TOTAL study [3]. Patient, coronary obstruction, intervention, necrosis, and consecutive inflammation-related factors like atherosclerotic risk factors, thrombus burden, distal embolization, microvascular obstruction (characterized by TIMI flow and MBG), and inflammation markers have already been investigated in thrombus aspiration studies. However, the question is still unanswered as to why thrombus removal is not beneficial regarding short- and long-term mortality [4]. The mortality of myocardial infarction shows a close correlation with thrombus burden and microvascular obstruction parameters. Thrombus aspiration obviously improves microvascular perfusion and reduces thrombus burden. However, it is still not clear why thrombus aspiration fails to improve mortality in clinical studies. The age of the clot as a predictor is an interesting, however not deeply investigated, issue. In thrombus aspiration studies released to date, routine laboratory and patient-related parameters are not described and compared according to the thrombus age.

Thrombectomy has been a useful tool to investigate the structure of coronary thrombi and to study the thrombus components during its evolution. There are still many unresolved questions regarding the association of time and thrombus architecture and the presence of different components at different stages of thrombus evolution during myocardial infarction (MI). Analysis of coronary thrombi with advanced imaging techniques has become a major research focus area for a decade, and some interesting results have been described already. In most studies, the structure of coronary thrombi was investigated by hematoxylin–eosin (H&E) staining or immunostaining for fibrinogen, platelets, and leukocytes. According to the results of these studies, coronary thrombi are mainly composed of fibrin, platelets, erythrocytes, cholesterol crystals, and leukocytes, with fibrin being the predominant component [5,6,7].

Thrombus formation is a dynamic process regulated by flow, blood cells, and plasma proteins [8]. The ischemic time is supposed to have an impact on thrombus composition. It was found earlier that the fibrin content increased and platelet content decreased over time, indicating a fast-evolving process during acute coronary occlusion [5]. It is obvious that coagulation plays an important role in coronary thrombus formation. However, the presence of its factors (beyond fibrinogen) was hardly investigated.

Among clotting factors, factor XIII (FXIII), a zymogenic transglutaminase, is involved in the final phase of coagulation. In the plasma, it circulates in complex with fibrinogen as a heterotetramer of two catalytic subunits A and two carrier/inhibitory subunits B. The FXIII-A subunit is also present in different cells, including megakaryocytes, platelets, monocytes, macrophages, and osteoblasts [9]. After activation by thrombin, FXIII-B leaves the complex, while the activated FXIII-A (FXIIIa) binds to fibrin and crosslinks fibrin γ- and α-chains; in this way, it increases the stiffness of the fibrin meshwork and the resistance to fibrinolysis. Moreover, part of the antifibrinolytic effect of FXIIIa is mediated by crosslinking α2-plasmin inhibitor (α2-PI), a serine protease inhibitor, to fibrin α-chains. α2-PI, which is the main physiologic inhibitor of plasmin, remains fully active after crosslinking and protects the forming clot from spontaneous fibrinolysis [10]. Their appearance and their relation in the coronary thrombus have not been investigated to date.

Different studies suggested the major role of neutrophil extracellular traps (NETs) in thrombus stabilization and their presence in coronary thrombus [11,12]. It was shown that the NET content of thrombi varies at different arterial locations, and in the coronary vessels, it is 2.5-fold higher as compared to the peripheral arteries [13]. The main compositions of NETs are the decondensed DNA backbone and histones, and their primary function is to trap and kill bacteria in the case of inflammation [14]. However, bacterial invasion is not the only inducer of NET formation and NETosis is considered as a consequence of cell death. The NET formation also plays a role in sterile inflammatory diseases, such as atherosclerosis [15]. It was demonstrated earlier that NETosis can promote coagulation; thus, it may contribute to thrombosis. NETs were found to be associated with tissue factor, factor XII, factor X, activated FVII, and also with a natural anticoagulant protein, activated protein C (APC) [16]. Protein C (PC) is activated by thrombin in the presence of thrombomodulin (TM) and endothelial protein C receptor (EPCR), a cell surface protein, which binds PC through its Gla domain and presents it to the thrombin-TM complex. APC inactivates active factor VIII and V [17]. APC/PC is also known as a cytoprotective molecule, which prevents cellular and organ vascular injuries [18]. Extracellular histones—products of NETosis—were shown to contribute to endothelial dysfunction, organ failure, and death during sepsis, and can be targeted therapeutically, particularly by APC [19]. It was demonstrated that APC induces heme-oxygenase-1 in macrophages, resulting in thrombus resolution. The appearance of APC/PC in the coronary thrombus and its relation with NETs is not known.

Evaluating aspirated coronary thrombi specimens requires advanced imaging technologies, which allow the analysis of the composition and structure of the coronary thrombus. The primarily used advanced techniques in this field are spectral histopathology, multiphoton microscopy, and high-resolution microscopy, such as electron microscopy (transmission electron microscopy, scanning electron microscopy) and confocal microscopy [20]. In the current study, coronary thrombi sections that have been aspirated and stained were subjected to microscopic examination using confocal laser scanning microscopy analysis, as many others have already chosen this type of examination and published their results earlier [8,13,21,22,23].

Our aims were—in the first part of our study—to investigate routine laboratory parameters and patient-related factors in the mirror of the thrombus age. We aimed to investigate whether the thrombus age showed an association with laboratory and clinical factors previously investigated in different studies and with mortality. Moreover, we aimed to characterize the elements of coronary thrombi focusing on the appearance of APC/PC, the factors closely related to fibrinogen-fibrin (FXIII and α2-PI), and the relation of APC/PC to the elements of NETs. We intended to examine if differences could be observed in the appearance of these elements according to the different stages of thrombus organization. We describe in this paper the structural features of coronary thrombi of different ages, focusing on the presence and localization of the above-mentioned factors.

## 2. Results

### 2.1. General Characteristics of the Study Population

In the study period, *n* = 164 patients underwent coronary thrombus aspiration; among them, there was male dominance: *n* = 115 males and *n* = 49 females were treated. The mean age of the patients was 61.1 ± 11.9 years. The patients were subclassified according to the background of their coronary occlusion, where *n* = 125 had atherosclerotic coronary plaque rupture-related native coronary artery occlusion (AC), *n* = 18 had a coronary embolism, *n* = 8 had occlusion of the saphenous coronary graft (SVG), and *n* = 13 had stent thrombosis. Due to the different clinical and pathophysiologic nature of the coronary occlusion, coronary embolism, SVG, and stent thrombosis-related cases were excluded from the present study.

### 2.2. Investigation of Routine Clinical, Laboratory, and Angiographic Indices

Within the group of coronary plaque rupture-related cases, there was no difference in age, gender, and in classical risk factors (hypertension, diabetes mellitus, hyperlipidemia, and smoking) among acute, subacute, and late-comers (Table 1).

The pre-procedure ejection fraction, TIMI flow, white blood cell, neutrophil cell and platelet counts, and the eGFR were also comparable within the three subgroups, while the monocyte count was the highest in late-comers. Gradual and inverse relationships could be observed in post-procedural TIMI flow and in MBG values as a function of the time elapsed after coronary occlusion. The CRP level showed a gradual increase with the time elapsed after coronary occlusion. CK values at admission—as it was expected—were the highest in subacute cases and showed only a slight elevation in the acute phase. While thrombus mass was the highest in late-comers, prognosis one year after PCI and thrombus aspiration did not depend on the time elapsed after coronary occlusion.

We investigated clinical and laboratory markers, which could contribute to the bad prognosis indicated by one-year mortality after PCI (Table 2).

From the whole population, we had data about the one-year survival of *n* = 121 patients, among whom *n* = 17 did not survive one year after PCI. Older age, diabetes mellitus, lower ejection fraction, poor post-procedural TIMI flow and MBG, higher white blood cell, neutrophil and platelet counts, lower eGFR, higher CK at admission, and higher weight of aspirated thrombus were associated with one-year mortality. Among them, the presence of a high aspirated thrombus mass (i.e., above 20 mg), diabetes mellitus, and higher neutrophil cell count were independent predictors of mortality in the adjusted model. A higher ejection fraction at admission was independently associated with a better prognosis (Table 3). Thrombus mass below 20 mg showed a good negative predictive value for one-year mortality (NPV = 0.95), while the positive predictive value was low (PPV = 0.23), and the ROC analysis resulted in a 0.732 AUC value (95% CI 0.600–0.863, *p* = 0.002).

On the contrary, infarct-related artery (i.e., LAD, CX, or RCA) and the number of affected vessels (i.e., one-, two-, or three-vessel disease) did not show a significant correlation with prognosis (Table 2). The presence of procedural distal embolization was more frequent in those patients who did not survive. However, the difference in frequency was statistically not significant. The length and diameter of the implanted stent, length of LAD, and proximal or distal thrombus localization were not correlated significantly with one-year mortality.

Upon investigating the associations between aspirated thrombus mass and different clinical and laboratory factors, the age of thrombus (i.e., acute-subacute-late), CK level at admission, and infarct-related artery (IRA) showed a significant correlation (r = 0.278, *p* = 0.002 for thrombus age; r = 0.288, *p* = 0.001 for CK; and r = 0.227, *p* = 0.011 for IRA). There was a tendency toward higher CRP levels in the case of a higher aspirated thrombus mass (r = 0.175, *p* = 0.059).

### 2.3. Determination of the Thrombus Age by Classical Histopathology

Based on the well-known dynamic development process of coronary thrombosis, we examined the aging of the aspirated thrombi by H&E staining and categorized them according to fresh, lytic, or organized. Thrombus age was classified based on the previously described criteria [24,25,26,27].

**Figure 1:**
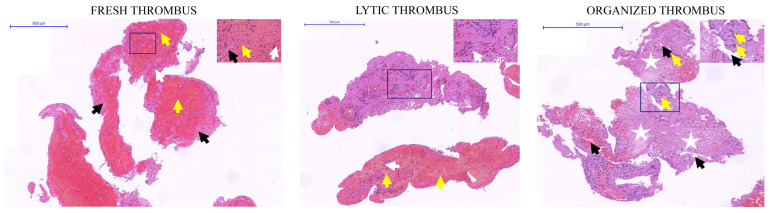
Representative images of hematoxylin-eosin (H&E) staining at the different stages of thrombus evolution (fresh, lytic, and organized). The magnified area showed the well-characterized elements of the different stages of thrombus evolution, in concrete terms, intact cellular elements and layered structures of the fresh thrombus (fibrin: black arrows, erythrocytes: yellow arrows, leukocytes: white arrows). In the lytic thrombus: colliquation (yellow arrows), and karyorrhexis of granulocytes (cellular debris: white arrows). In the organized thrombus: internal growth of smooth muscle cells, connective tissue deposition, and proliferation (extensive hyalinized areas with reduced cellularity: white stars, myofibroblast proliferation: yellow arrows, and recanalization: black arrows). Scale bar: 500 μm. Scale bar of the magnified area: 50 μm.

Fresh, lytic, and organized thrombi were found in 8 of 24 patients (33.33%) each. Thrombi classified into fresh, lytic, and organized by H&E staining corresponded to their pre-classification by clinical and laboratory indices in all cases. In the fresh thrombus, intact cellular elements and layered structures were observed. These were intracoronary masses with a heterogeneous structure composed of a fibrin network along with intact blood cells, such as granulocytes, platelets, and erythrocyte aggregates. In the lytic thrombus lysis, the colliquation and karyorrhexis of granulocytes were detectable. These are characterized by cell apoptosis and areas of coagulation necrosis associated with the redistribution of cellular elements. The appearance of macrophages and a tendency of granulocytes to polarize with lytic activity on the surface of the fibrin network was observed. In the organized thrombus highly structured organization, internal growth of smooth muscle cells and connective tissue deposition, and proliferation were the main histopathological characteristics. The internal accumulation of fibroblasts and deposition of loose connective tissue were observed (Figure 1).

The thrombi, however, were not homogeneous, as they often showed features of organization, lytic changes, and elements of fresh thrombus in the same specimens simultaneously. In order to examine the presence of the hemostasis factors in the thrombi and compare their appearance according to the thrombus age, we applied age-specific region of interest (ROI) upon analysis.

### 2.4. The Appearance of Hemostasis Proteins and Markers of NETs in Coronary Thrombi at Different Stages of Evolution

After the initial processing of *n* = 164 patient samples, a detailed analysis of coronary thrombi was performed in *n* = 24 male patients suffering from atherosclerotic coronary plaque rupture-related native coronary artery occlusion (AC) by using immunofluorescent staining and confocal microscopic analysis. By exclusively including male patients, the current study effectively eliminates gender-related variables, enhancing the reliability of its findings.

**Figure 2:**
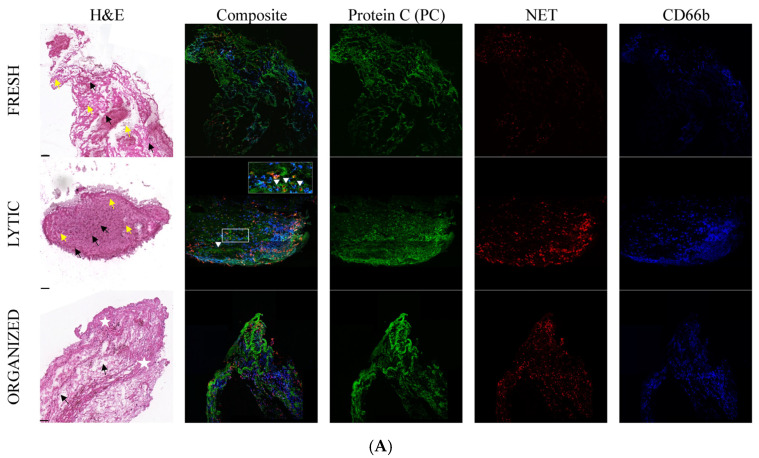

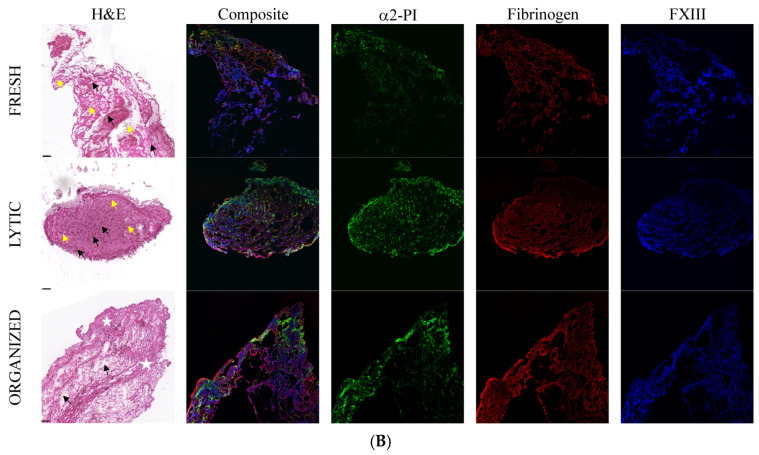
(**A**) Collection of the representative immunofluorescent imaging in aspirated coronary thrombus. The labeled markers were APC/PC, NET, and CD66b. White arrowheads show the formation of a nest-like structure by APC/PC around the elements of the NET. The morphological changes in the histological images are described in Figure 1. In the fresh thrombi, black arrows show an inhomogeneous structure with a fibrin network, and yellow arrows show cellular components of blood. In the lytic thrombi, yellow arrows demonstrate a tendency to homogenization with colliquation, and black arrows show fragmentation of nuclear elements. In the organized thrombi, white stars indicate an inhomogeneous structure with hyalinized areas, and black arrows indicate the area of recanalization. Images were taken with the Olympus FluoView 3000 confocal microscope and 3DHISTECH Pannoramic SlideViewer. The scale bar in the HE images is also applicable to the fluorescence images. Scale bar: 100 μm. The magnified area is 20×. (**B**) Collection of the representative immunofluorescent imaging in the aspirated coronary thrombus. The labeled markers were α2-PI, Fibrinogen, and FXIII. The morphological changes in the histological images are described in Figure 1. In the fresh thrombi, black arrows show an inhomogeneous structure with a fibrin network, and yellow arrows show cellular components of blood. In the lytic thrombi, yellow arrows demonstrate a tendency to homogenization with colliquation, and black arrows show fragmentation of nuclear elements. In the organized thrombi, white stars indicate an inhomogeneous structure with hyalinized areas, and black arrows indicate the area of recanalization. Images were taken with the Olympus FluoView 3000 confocal microscope and 3DHISTECH Pannoramic SlideViewer. The scale bar in the HE images is also applicable to the fluorescence images. Scale bar: 100 μm.

The fresh thrombi extracted from *n* = 8 patients exhibited a unified, layered structure, which had a very tiny appearance of the NET marker (citrullinated H3 histone) and the CD66b marker (Figure 2A).

**Figure 3:**
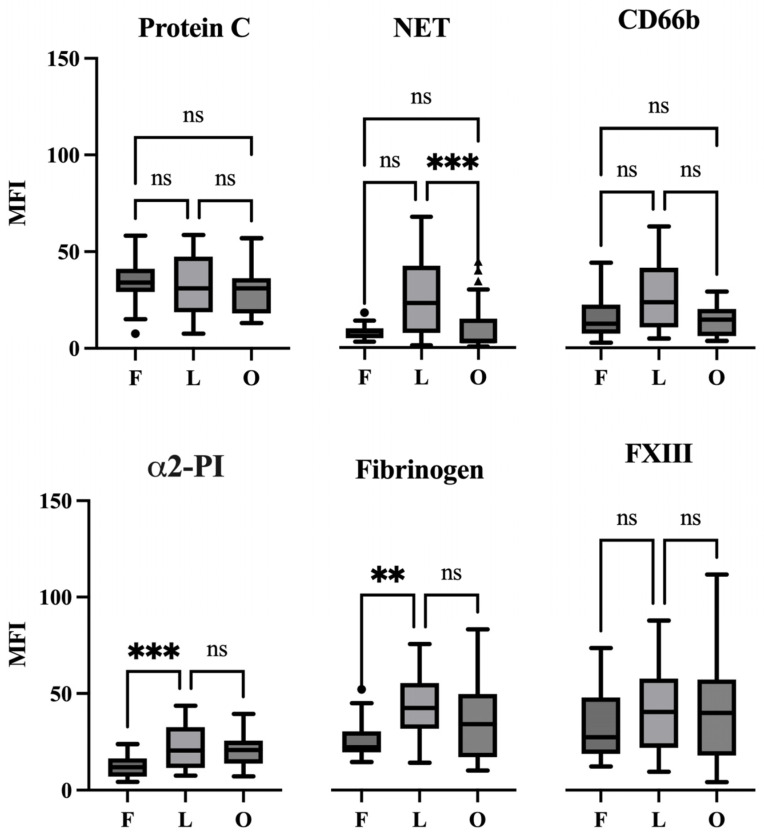
Boxplots showing the MFI values of the detected marker over the fresh, lytic, and organized stages of aspirated coronary thrombi. *p* < 0.05. Abbreviations: fresh (F), lytic (L), and organized (O) stages of aspirated coronary thrombi. (ns, not significant; ** *p* < 0.01; *** *p* < 0.001).

More specifically, the fresh thrombi had the smallest proportion of NET markers with a mean fluorescence intensity (MFI) of 8.176 ± 0.72 (mean ± SEM) (Figure 3).

Similarly, the fresh coronary thrombi exhibit a low level of α2-PI; the MFI of this marker was 12.31 ± 1.22. MFI values for PC/APC, CD66b, fibrinogen, and FXIII were 34.3 ± 2.26, 16.1 ± 2.28, 26.5 ± 2.24, and 33.4 ± 3.68, respectively. In terms of morphological appearance, α2-PI showed a punctiform structure (Figure 2B), which was also seen in the case of NET and CD66b (Figure 2A).

As expected, fibrin showed a well-recognizable composition already in the early phase of the thrombus formation. The level of APC/PC and FXIII markers were also considerable in the fresh thrombi. Fibrin fibers appeared as a scaffold filling the whole thrombus. FXIII was detected at the same localization appeared as a non-punctiform but rather continuous structure alongside fibrin, and the analysis of co-localization showed a strong correlation with fibrin (Pearson correlation coefficient (PCC) was 0.65 ± 0.01). The APC/PC occupied the entire thrombus area and appeared as a non-punctiform structure.

The intensity of the investigated markers changed markedly in the next stage of thrombus evolution (lytic phase). The MFI values of NET marker (25.8 ± 3.72), CD66b (27.0 ± 3.56), and α2-PI (22.6 ± 2.41) increased, and the difference was statistically significant in the case of α2-PI as compared to its amount in the previous phase. The amount of fibrin (43.15 ± 3.36) was also highly increased as compared to the fresh thrombi. The amount of FXIII (42.1 ± 4.6) slightly increased in lytic thrombi, while the APC/PC intensity (32.3 ± 3.36) was constant. The appearance of α2-PI and NET was similar to in the fresh thrombi, showing a punctiform manner with a non-homogeneous distribution. APC/PC in this age of thrombus showed a honeycomb-like structure, and also CD66b appeared similarly in a large area of the thrombi, suggesting that they close round the NET (please see arrow in Figure 2A). The APC/PC-NET co-localization was considerable (PCC was 0.49 ± 0.02). The fibrin-FXIII co-localization maintained a persistent and strong correlation (PCC was 0.69 ± 0.01) in lytic thrombi.

In the organized thrombi, the amount of NET (9.96 ± 2.76) and CD66b (14.0 ± 1.84) markedly decreased as compared to the previous phase in the case of the NET marker, and the decrease in MFI values was statistically significant (*p* = 0.0007). The amount of investigated hemostasis markers remained similar to those in the lytic phase. The MFI values of PC/APC, α2-PI, fibrinogen, and FXIII were 30.8 ± 2.68, 21.2 ± 1.74, 37.0 ± 4.35, and 41.2 ± 5.37, respectively. The appearance of α2-PI and NET was similar as in the previous phases of thrombus evolution, showing a punctiform manner and a non-homogeneous distribution. APC/PC and CD66b appeared in their honeycomb-like structure; however, as the whole structure of thrombi became looser, the immunopositive area also decreased. As the amount of both NET and CD66b decreased markedly, the investigation of their co-localization with APC/PC could not be conclusive. Fibrin structure—as it was expected—became fragmented in the organized thrombus. FXIII remained strongly associated with fibrin, and PCC was 0.63 ± 0.01.

## 3. Discussion

In our study, we investigated laboratory and patient-related factors in patients with different stages of thrombus formation during the time course of STEMI. The highest CK values, as expected, were registered in the subacute phase of STEMI, while CRP and WBC count, especially monocyte count, gradually increased across the phases of STEMI, demonstrating the development of the inflammatory reaction. Thrombus burden and microvascular reperfusion parameters showed an unfavorable tendency across age categories and were the most disadvantageous in late-comers. However, one-year mortality across the three groups did not show a significant difference in our patient population. It was demonstrated earlier that a high thrombus burden was a significant predictor of distal embolization, which was associated with a bad prognosis after PCI [28,29]. However, we found no significant predictors of distal embolization among our investigated indices in the whole study group. The most likely variable, which is expected to show an association with distal embolization, is the thrombus mass in our study; however, it also did not show a significant correlation with distal embolization (r = 0.132, *p* = 0.148). There was also no significant correlation among acute, subacute, or late-comers with distal embolization. Post-procedural TIMI and MBG were, as expected, unfavorable in our patients with distal embolization (r = −0.183, *p* = 0.041 for TIMI and r = −0.181, *p* = 0.044 for MBG, respectively). The frequency of distal embolization was higher in our patients who were deceased at one-year follow-up, but the difference in frequency did not reach statistical significance. This may be due to our small sample size in the non-survival group. A higher thrombus mass (i.e., above 20 mg) was significantly associated with one-year mortality in our patients, and—in addition to diabetes mellitus, low EF, and higher WBC (neutrophil)—it was shown to be an independent risk factor of one-year mortality.

**Figure 4:**
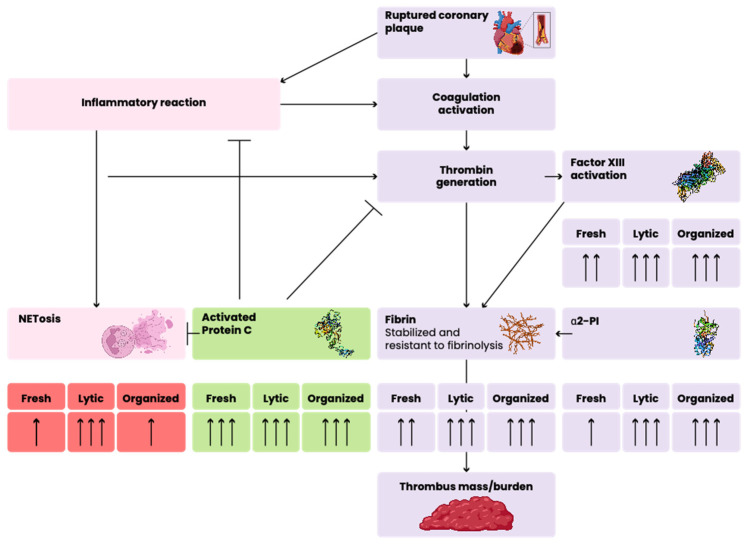
Schematic representation of the association of different elements of hemostasis in thrombus evolution. Number of arrows (one–two–three) under “fresh, lytic, and organized” status represents the amount of the investigated marker in the extracted coronary thrombus at the given stages. Arrows with triangular end and arrows with horizontal end represent positive and negative associations, respectively.

In this study, we investigated the structure of the aspirated coronary thrombi of patients with atherosclerotic coronary plaque rupture associated with myocardial infarction. Based on the classic histopathology features, the thrombi were classified into fresh, lytic, and organized categories. In this paper, we described the appearance of FXIII, α2-PI, and APC/PC at the different stages of thrombus evolution, which have not been described earlier, although being essential factors from the point of view of thrombus strength and fibrinolysis. APC/PC has an important role not only in coagulation–anticoagulation but also in anti-inflammatory and cytoprotective processes. We determined the associations of FXIII and α2-PI with each other and with fibrinogen, a well-studied component in the coronary thrombus. Moreover, an association of APC/PC with the elements of NET and with activated granulocytes was also studied in this paper (Figure 4).

Fibrin showed a usual structure in coronary thrombi as described in previous studies [5,8,20,30,31,32,33]. It is well-known that crosslinked fibrin fibers are one of the main components of thrombi, and the amount of fibrin shows an increase over time during thrombus evolution until the lytic stage; then, by organization, its amount slightly, but not significantly, decreases. This was also seen in our cases. FXIII is intimately associated with fibrin by playing a pivotal role in the crosslinking procedure as a transglutaminase. In our investigated coronary thrombi, the amount of FXIII was high, and it did not change significantly during their evolution. In line with its known biochemical role, the co-localization of FXIII and fibrin was the highest among all investigated markers in all stages of thrombus evolution, and the visual appearance of FXIII tightly and continuously followed the fibrin structure.

Not only FXIII but also α2-PI play elemental roles in fibrinolysis regulation. α2-PI, a serine protease inhibitor, is crosslinked to fibrin by FXIII, stabilizing the fibrin clot and inhibiting fibrinolysis [34]. Indeed, the significance of α2-PI in the context of coronary thrombosis remains largely unexplored. To date, the appearance and the relation of α2-PI in coronary thrombus have not been investigated. In in vitro clotting studies using purified fibrinogen, FXIII, and α2-PI after thrombin activation, the crosslinking of α2-PI to α-chain of fibrin proceeds very rapidly and is almost completed before the formation of the γ-chain dimers and α-chain polymers of fibrinogen [35]. However, much is yet unknown about the process of crosslinking in vivo. In our study, the amount of α2-PI was low in the fresh thrombi, and we observed a statistically significant increase from the fresh to the lytic phase of coronary thrombus, which was parallel with the increase in fibrin. The MFI values of α2-PI did not increase further in the organized phase. Opposite to the architecture of FXIII, the appearance of α2-PI was rather discrete but evenly distributed and consistently associated to fibrin. This discrete distribution may be explained by the in vitro observation, according to which about 30–50% of the α2-PI in the plasma binds to the clot, which means that one molecule of α2-PI is found in the clot for every 15–25 molecules of fibrin. Alternatively, FXIII may crosslink α2-PI at dedicated points to fibrin, which has not been explored as yet.

Based on our observations and the above-mentioned biological roles of FXIII and α2-PI in thrombus formation and stabilization, it seems to be beneficial to test these factors as therapeutic targets in coronary atherosclerosis for the prevention and treatment of STEMI in addition to standard patient care.

Previous studies have demonstrated that APC is a crucial regulator of fibrinolysis and coagulation, and it has not only anticoagulant but also anti-inflammatory and cytoprotective properties on a variety of cell types, including monocytes and neutrophils [18,36,37,38,39,40]. It was suggested that APC’s cytoprotective and anti-inflammatory actions might include the regulation of neutrophil function and NET formation based on the findings that APC could cleave extracellular histones in animal models [41]. The presence of NET in thrombi of different origins—including aspirated coronary thrombi—has already been described [42,43,44,45,46,47,48,49]. CD66b, a member of the carcinoembryonic antigen family, could potentially play an important role in the NET’s formation procedure. Yunoki et al. observed a significant amount of CD66b immunopositive area in an aspirated thrombus, which could relate to an impaired coronary microcirculation [50]. The concept of thrombo-inflammation was raised several years ago, and the interplay between the two basic mechanisms in the development of cardiovascular diseases, including myocardial infarction, was demonstrated [51]. Neutrophils and NETs are localized in all types of complicated lesions, suggesting an association of their presence with acute complications of atherosclerosis [43]. APC was shown to play a pivotal role as a cardio-protective factor not only via its anticoagulant but also via its anti-inflammatory and cytoprotective features [52]. The relationship between APC and the elements of NET was first investigated in aspirated coronary thrombus in our study. In our experiments, a relatively high amount of APC/PC was detected in all stages of thrombus evolution, suggesting that APC/PC appears at a very early stage of thrombus formation. The activation of PC by thrombin is an essential step in the natural anticoagulant processes and the action of APC by inactivating activated FVIII and FV limits thrombin generation, and thus, fibrin formation. APC can also promote fibrinolysis, further limiting thrombus growth. By cleaving histones of NETs, APC may decrease the inflammatory and cytotoxic reactions associated with acute atherosclerotic plaque rupture. Taking these actions together, the presence of APC in coronary thrombus formation is suggested to be a protective factor, which is worthy of further investigations both in in vitro and in clinical studies in order to consider it as a potential therapeutic use.

We found a very small amount of the NET marker in the fresh thrombi and during evolution; the intensity values of NETs were remarkably increased by the lytic phase. Our results demonstrate that fresh thrombi have a slowly starting NETosis process, which is consistent with the current knowledge of thrombus formation. In the organized stage, however, the amount of NET decreased markedly. During the lytic phase, where a higher amount of NET marker was present, a considerable level of co-localization was observed in relation to APC/PC and the NET marker citrullinated H3 histone (PCC was 0.49 ± 0.02). This observation raises the hypothesis that, after NET formation, APC takes part in histone degradation, leading to a decrease in NET, thus trying to eliminate its adverse consequences. The amount of CD66b followed the dynamics of citrullinated H3 histones’ appearance, i.e., there was only a small amount of this marker in the fresh thrombus, which highly increased in the lytic phase and thereafter decreased. It is surmised that the appearance of granulocytes after plaque rupture results in NET formation in the growing thrombus. The limitation of NET presence in the organized thrombus may be due to the lower amount of granulocytes present in this stage. Taking our observations together, we can conclude that, after a slow NETosis leading to the highest amount in the lytic stage of coronary thrombus, the amount of NETs is decreased in part due to the lower number of granulocytes that produce them, especially due to the action of histone degradation at least partly by APC. This hypothesis is supported by the visual appearance of these markers in the lytic phase. APC/PC forms a nest-like structure closes round the elements of NET.

It is difficult, however, to draw a clear conclusion about the events in the different stages of thrombus evolution because it is difficult to separate the different stages of thrombus evolution from each other in the ex vivo experiments since intracoronary thrombi aspirated in STEMI frequently show more than one stage of maturation [27]. However, this exploratory study could set the essential starting point for future translational medicine studies by identifying basic mechanisms and key variables. Although the impact of the current study is largely theoretical, these findings could be crucial for guiding further clinical research in the field of coronary thrombosis.

Thrombus aspiration during pPCI is no longer part of the clinical routine [3], which makes future experiments cumbersome. In the studies executed to date, the aspirated thrombus weight has not been measured and only a rather subjective semi-quantitative assessment has been conducted based on coronary angiogram. In our study, the weight of aspirated thrombi was measured, and it showed a gradual increase as a function of the time elapsed after coronary occlusion. A higher thrombus weight correlated significantly with one-year mortality.

Our study has limitations that should be acknowledged. This was a single-center analysis with a limited sample size, although the sample size was comparable to that of samples in similar pathological studies. During thrombus aspiration, the structure might suffer distortion, and the thrombus surface and core, tail, and head could not be differentiated. We did not investigate the association of thrombus composition with clinical prognosis after pPCI due to the limited sample size and a rather similar appearance of thrombi in the same age category. Future studies using different methods, such as high-resolution nuclear magnetic resonance (NMR), mass spectrometry, and tissue microarrays (TMAs), could provide a more detailed spatial distribution of protein expression in the thrombus microenvironment. These techniques have enabled in-depth quantitative tissue mapping to examine inter-sample and intra-sample differences in the aspirated coronary thrombi. We were also not able to investigate the potential role of prior treatment with anti-platelet or anticoagulant drugs in thrombus composition since only a minority of patients were on aspirin before the diagnosis of STEMI, and no other anti-thrombotic therapy was used as primary prophylaxis.

## 4. Materials and Methods

### 4.1. Patients

The Cardiology Clinic of the University of Debrecen is a large referral hospital with an annual primary PCI (pPCI) volume of around 700–750 procedures. Patients (*n* = 164) between January 2012 and December 2018 were originally enrolled in the study who had STEMI confirmed by ≥0.2 mV ST-segment elevation in two or more contiguous leads within 48 h after the onset of symptoms and were eligible for PCI. Before a pPCI 5000 IU of heparin, a loading dose of the aspirin (250–500 mg) and clopidogrel (600 mg) was administered. Two different thrombus aspiration systems were used: QuickCat (The Spectranetics Corporation/Philips Healthcare, Colorado Springs, CO, USA) and ASAP (Merit Medical Systems, Inc., South Jordan, UT, USA). The aspiration devices used were 6F compatible, with an extraction lumen area of 0.86 mm^2^ in the case of QuickCat and 1.0 mm^2^ in the case of the ASAP device. The aspiration efficacy is known to be determined mainly by the extraction lumen area, which did not show a major difference between the two types of devices. The use of the aspiration device was at the discretion of the operator. The aspirated thrombi were processed if a sufficient amount was available for further analysis. The weight of each thrombi was measured before being embedded into Thermo Scientific Shandon Cryomatrix Frozen Embedding Medium (Thermo Shandon, Pittsburg, PA, USA) and frozen by liquid nitrogen. Thereafter, the specimens were stored at −80 °C until analysis. Another part of the thrombus samples was fixed and embedded into paraffin. Patients with coronary thrombi originating from coronary embolism (*n* = 18), saphenous coronary graft (SVG, *n* = 8), and stent thrombosis (*n* = 13) were excluded at the first step of recruitment, since the investigation of thrombi related to plaque rupture of the native coronary artery was the focus of the present study. Sample size calculation was conducted based on the thrombus mass and survival rate (which is around 0.1 according to our national infarction registry), where (0.05 as type I error rate and 0.80 as statistical power) risk ratio was set at 3.5 and drop rate was set at 15%. By this calculation, the sample size was *n* = 60 in both groups (i.e., patients with extracted thrombus mass below and above 20 mg). Based on this, we included consecutive patients having a low and high thrombus mass *n* = 65 and 60, respectively, in our study. Clinical indices—like age, gender, risk factors of atherosclerosis, body mass index (BMI), pain to thrombus aspiration time—and laboratory indices—like blood cell counts, kidney function (glomerular filtration rate, eGFR), and C-reactive protein (CRP) levels—were obtained from our medical database. Serum creatinine (for eGFR calculation), CRP, and creatine-kinase (CK) were measured by Roche reagents on a Cobas analyzer (Roche, Mannheim, Germany). Blood count was investigated by Sysmex hematology analyzer (Sysmex, Kobe, Japan). Angiographic indices like an extension of coronary artery disease, pre- and post-TIMI flow (i.e., thrombolysis in myocardial infarction flow), lesion length and diameter, the occurrence of distal embolization, and myocardial blush grade (MBG) of infarct-related artery (IRA) were collected from an electronic database and by the visual re-evaluation of coronary angiographies. The TIMI flow grade was defined as 0 if there was no anterograde flow beyond occlusion, grade 1 was defined if the contrast penetrated the occlusion but the distal parts failed to opacify the entire coronary, grade 2 was defined as partial perfusion and complete opacification of the coronary bed but slower distal clearance of the contrast material, and grade 3 was defined as complete perfusion and anterograde flow with rapid clearance distally to the occlusion. MBG−0 was defined as the failure of the contrast material to enter the microvasculature, resulting in no ground glass appearance (“blush”), indicating a lack of perfusion at the tissue level. MBG−1 was defined as if the dye slowly entered but failed to exit the microvasculature (dye was present more than 30 s in the myocardium); MBG−2 meant a delayed enter and exit of the contrast material from the microvasculature, and contrast persisted after three cardiac cycles but washed out within 30 s; and MBG−3 meant a normal enter and exit of the dye from the microvasculature, and it completely cleared within three cardiac cycles from the tissue. MI was classified as acute, and the aspirated thrombus was suspected as fresh if less than 12 h elapsed between the onset of the chest pain and thrombectomy and/or creatine kinase (CK) level at admission was less than two times of upper limit of normal. Thrombi aspirated between 12 and 24 h and more than 24 h after onset of chest pain and/or CK level at admission was more than two times of the upper limit of normal or peaked at admission were classified subacute and late-comer, respectively.

Informed consent from the patients was acquired, although thrombus aspiration was part of the clinical routine. The work was carried out according to the principles laid down in the Declaration of Helsinki and amended in 2008 by the World Medical Assembly in Seoul, Korea. Ethical approval for the study was obtained from the National Ethical Council (3166/2012/HER).

### 4.2. Methods

After aspiration, one part of the thrombus samples was fixed and embedded into paraffin. Sections were cut at 5μm thickness in a series (Cryostat, Leica CM1860 UV, Nussloch, Germany). These samples were used for the examination of morphological characteristics; therefore, they were stained with H&E and reviewed and evaluated by an independent pathologist.

The other part of the thrombus samples was deep frozen in liquid nitrogen and stored at −80 °C until multiple immunofluorescent staining and confocal laser scanning microscopy analysis. In these circumstances, consecutive 5μm slices of all thrombi specimens were multiple stained against fibrinogen, FXIII, and α2-PI or APC/PC, histone H3, and CD66b. The following antibodies and dilution ratios were used: mouse monoclonal anti-human α2-PI antibody conjugated with FITC, dilution ratio: 1:100 (in-house developed, reacting with all forms of α2-PI); polyclonal rabbit anti-human fibrinogen antibody (ab34269,Abcam, Cambridge, UK), dilution ratio: 1:100 used with Alexa Fluor^®^ 568-labelled goat anti-rabbit IgG (H + L), dilution ratio: 1:500 (A11011); mouse monoclonal anti-human FXIII-A subunit antibody conjugated with Alexa Fluor^®^ 647 (developed in-house), dilution ratio: 1:500; monoclonal mouse anti-human Protein C (AHPC−5071, Haematologic Technologies/CellSystems, Troisdorf, Germany), dilution ratio: 1:500; Dylight 488 anti-mouse IgG (DI-2488, Vector Laboratories, Newark, NJ, USA), dilution ratio: 1:500; polyclonal rabbit anti-histone H3 (citrulline R2 + R8 + R17) antibody–ChIP Grade (ab5103, Abcam, Cambridge, UK), dilution ratio: 1:400; and goat anti-rabbit IgG lexa Fluor^®^ 568 and monoclonal mouse anti-human CD66b conjugated with Alexa Fluor^®^ 647 (305109, BioLegend, San Diego, CA, USA), dilution ratio: 1:100. All sections were coverslipped with a Hydromount water-based mounting medium (NAT1324, National Diagnostics/ Scientific Laboratory Supplies, Nottingham, UK). The deep-frozen thrombi specimens were also stained with H&E and reviewed and evaluated by an independent pathologist.

Images were acquired by Olympus FluoView 3000 confocal microscope (Olympus, Tokyo, Japan), and its multi-area time-lapse software module was used for image analysis. The confocal images of fresh, lytic, and organized thrombi were captured using the same laser intensity and detector sensitivity settings to ensure reliable and consistent MFI measurements. The H&E slides were digitalized with a Pannoramic Confocal microscope and were analyzed (including morphometric measurements) with Pannoramic SlideViewer (version 2.3) (3 DHISTECH, Budapest, Hungary). For the confocal image analysis of the coronary thrombi specimens, the open-source software Fiji (version 2.3) (Fiji Is Just ImageJ) [53] was used. For the quantification of the different characterized elements of the confocal fluorescent images, a standardized, previously well-detailed, published methodology was adapted [54]. The results of the co-localization analysis were defined as a Pearson correlation coefficient as implemented by the JaCoP plugin in Fiji.

### 4.3. Statistical Analysis

The distribution of the continuous variables was examined by the Kolmogorov–Smirnov test. In the case of normal distribution, the continuous variables (age) were expressed as mean ± SD, while they were expressed as median and interquartile ranges (IQR) in the case of non-normal distribution (BMI, ejection fraction (EF), white blood cell, neutrophil, eosinophil, monocyte, platelet counts, eGFR, CRP, and CK). The differences in the distribution among patients’ categorical variables were examined by using the χ2 test. Differences in the continuous variables among the different patients’ groups were analyzed by t-test statistics, ANOVA or by Mann–Whitney U test and Kruskal–Wallis test. Pearson or Spearman correlation analysis was performed according to the distribution of parameters. A logistic regression model was used for investigating the association of clinical and laboratory factors with one-year mortality after thrombus aspiration. The statistical analysis was performed using the Statistical Package for Social Sciences (SPSS version 28.0) software, Chicago, IL, USA. A *p*-value of 0.05 or less was considered to indicate statistical significance.

## 5. Conclusions

Generally, we can conclude that, by the evaluation of coronary thrombi, delicate, constant structural changes are observed during their age evolution. The appearance of FXIII closely follows the structure and amount of fibrin, while the amount of α2-PI is lower and it rather shows a discrete and consistent association to fibrin. Since both FXIII and α2-PI are pivotal factors in thrombus stabilization and fibrinolysis, it seems to be beneficial to test them as therapeutic targets in coronary atherosclerosis for the prevention and treatment of STEMI. PC/APC has a high amount in all stages of coronary thrombi and the close association with the elements of NET suggests its function in histone degradation. The protective role of APC in thrombo-inflammatory processes makes it a potential therapeutic agent in acute cardiovascular complications.

## Figures and Tables

**Table 1:** Detailed characteristics of patients with atherosclerotic coronary plaque rupture related to native coronary artery occlusion.

	Acute (<12 h), *n* = 69	Subacute (12–24 h), *n* = 28	Late (>24 h), *n* = 28	*p* Acute vs. Subacute	*p* Acute vs. Late	*p* Subacute vs. Late
Age (years), mean, SD	60.9 (11.4)	56.8 (10.3)	57.5 (12.1)	0.107	0.186	0.805
Gender (male/female), *n*	48/21	21/7	18/10	0.592	0.613	0.383
CK (U/L), median, IQR	180 (191)	1178 (682)	539.5 (1050)	<0.001	<0.001	0.069
One-year survival (frequency), %	87.9	85.7	81.5	0.754	0.403	0.671
Ejection fraction, median, IQR	47 (10)	42 (14)	48 (16)	0.120	0.767	0.205
TIMI flow pre-procedure (0–3 grade), *n*	47/10/7/5	20/3/3/2	22/3/2/1	0.969	0.766	0.890
TIMI flow post-procedure (0–3 grade), *n*	0/0/7/62	0/1/6/21	0/2/5/21	0.088	0.041	0.809
MBG (0–3 grade), *n*	3/12/17/37	4/5/11/8	5/5/5/13	0.075	0.171	0.314
Distal embolization yes/no, *n*	19/50	6/22	11/17	0.533	0.257	0.146
Thrombus mass (mg) median, IQR	17.1 (16.2)	18.7 (35.8)	35.0 (32.9)	0.136	<0.001	0.195
WBC (G/L) median, IQR	12.69 (6.16)	14.71 (4.80)	12.89 (4.56)	0.206	0.561	0.350
Neutrophil cell count (G/L) median, IQR	9.96 (6.04)	11.27 (5.62)	10.11 (4.48)	0.193	0.735	0.350
Monocyte cell count (G/L) median, IQR	0.63 (0.33)	0.81 (0.55)	1.06 (0.76)	0.010	<0.001	0.189
Eosinophil cell count (G/L) median, IQR	0.09 (0.17)	0.03 (0.21)	0.04 (0.07)	0.141	0.014	0.774
Platelet count (G/L) median, IQR	242 (96)	243 (82)	235 (76)	0.524	0.564	0.946
eGFR (mL/min/1.73 m^2^) median, IQR	84.5 (22)	90 (30)	88.5 (17)	0.146	0.626	0.323
CRP (mg/L) median, IQR	3.18 (5.46)	7.41 (27.41)	40.60 (106.1)	0.021	<0.001	0.003
Coronary occlusion site, IRA (LAD/CX/RCA), *n*	29/26/14	11/10/7	17/10/1	0.878	0.079	0.055
Smoking (frequency), %	66	80	77	0.216	0.339	0.789
Hypertension (frequency), %	65	71	75	0.555	0.349	0.763
Diabetes mellitus (frequency), %	33	32	25	0.814	0.631	0.554
Hyperlipidemia (frequency), %	48	43	41	0.617	0.497	0.874
BMI (kg/m^2^) median, IQR	28 (7)	30 (10)	29 (7)	0.396	0.305	0.791

CK, creatine kinase; IQR, interquartile range; WBC, white blood cell count; eGFR, estimated glomerular filtration rate; CRP, C-reactive protein; IRA, infarct-related artery; LAD, left anterior descendent coronary artery; CX, circumflex coronary artery; RCA, right coronary artery; BMI, body mass index; TIMI flow grade short definition: Grade 0 (no perfusion): There is no antegrade flow beyond the point of occlusion. Grade 1 (penetration without perfusion): The contrast material passes beyond the area of obstruction but fails to opacify the entire coronary bed distal to the obstruction. Grade 2 (partial perfusion): The contrast material passes across the obstruction with delay, but the filling of the distal territory is complete. Grade 3 (complete perfusion): Antegrade flow is normal and comparable to other non-obstructed vessels. MBG (myocardial blush grade, microvascular penetration of contrast material) short definition: MBG 0: Absence of myocardial blush or contrast density. MBG 1: Minimal myocardial blush or contrast density. MBG 2: Moderate myocardial blush or contrast density, but less than that obtained from the ipsilateral, non-infarct-related coronary artery. MBG 3: Normal myocardial blush or contrast density, comparable with that obtained from the ipsilateral, non-infarct-related coronary artery.

**Table 2:** Subgroup analysis of patients with atherosclerotic coronary plaque rupture-related native coronary artery occlusion according to one-year survival.

	Survivals, *n* = 104	Non-Survivals, *n* = 17	*p*
Age (years) mean, SD	58.1 (11.1)	64.3 (11.4)	0.034
Gender (male/female), *n*	75/29	9/8	0.112
Smoking (frequency), %	73	50	0.091
Hypertension (frequency), %	69.2	70.6	0.910
Diabetes mellitus (frequency), %	26.9	58.8	0.024
Hyperlipidemia (frequency), %	48.0	33.3	0.286
BMI (kg/m^2^) median, IQR	29 (7)	27 (9)	0.624
Time of occlusion (acute–subacute–late), *n*	58/24/22	8/4/5	0.722
CK (U/L) median, IQR	264.5 (805)	823 (2596)	0.010
Ejection fraction, median, IQR	48 (10)	35 (18)	<0.001
TIMI flow pre-procedure (0–3 grade), *n*	72/14/11/7	14/2/1/0	0.603
TIMI flow post-procedure (0–3 grade), *n*	0/1/12/91	0/2/6/9	<0.001
MBG (0–3 grade), *n*	4/18/29/53	8/3/2/4	<0.001
Coronary occlusion site, IRA (LAD/CX/RCA), *n*	28/8/68	7/1/9	0.485
WBC (G/L) median, IQR	12.89 (5.40)	16.95 (9.09)	0.001
Neutrophil cell count (G/L) median, IQR	9.73 (5.47)	13.73 (7.24)	<0.001
Monocyte cell count (G/L) median, IQR	0.68 (0.54)	0.88 (0.74)	0.199
Eosinophil cell count (G/L) median, IQR	0.05 (0.16)	0.015 (0.14)	0.097
Platelet count (G/L) median, IQR	237 (89)	261.5 (84)	0.050
eGFR (mL/min/1.73 m^2^) median, IQR	87.5 (14)	67.5 (39)	0.007
CRP (mg/L) median, IQR	4.99 (11.66)	11.12 (35.34)	0.585
No. of affected coronary arteries (1–3 vessel disease), *n*	50/36/18	7/6/4	0.794
Distal embolization frequency (% of pts) Stent length (mm) median, IQR Stent diameter (mm) median, IQR Length of LAD (1–3 categories), *n* Proximal/mid/distal thrombus localization, *n*	25.0 28.0 (26.0) 3.00 (0.5) 5/51/48 42/47/15	47.1 28.0 (26.0) 3.5 (0.5) 3/7/7 9/6/2	0.061 0.649 0.634 0.141 0.623
Thrombus mass (mg) Thrombus mass below and above 20 mg, *n*	18.2 (16.1) 58/46	41.7 (30.8) 3/14	0.002 0.004

CK, creatine kinase; IQR, interquartile range; WBC, white blood cell count; eGFR, estimated glomerular filtration rate; CRP, C-reactive protein; IRA, infarct-related artery; LAD, left anterior descendent coronary artery; CX, circumflex coronary artery; RCA, right coronary artery; BMI, body mass index; TIMI flow grade short definition: Grade 0 (no perfusion): There is no antegrade flow beyond the point of occlusion. Grade 1 (penetration without perfusion): The contrast material passes beyond the area of obstruction but fails to opacify the entire coronary bed distal to the obstruction. Grade 2 (partial perfusion): The contrast material passes across the obstruction with delay, but the filling of the distal territory is complete. Grade 3 (complete perfusion): Antegrade flow is normal and comparable to other non-obstructed vessels. MBG (myocardial blush grade, microvascular penetration of contrast material) short definition: MBG 0: Absence of myocardial blush or contrast density. MBG 1: Minimal myocardial blush or contrast density. MBG 2: Moderate myocardial blush or contrast density, but less than that obtained from the ipsilateral, non-infarct-related coronary artery. MBG 3: Normal myocardial blush or contrast density, comparable with that obtained from the ipsilateral, non-infarct-related coronary artery. Length of LAD defined as follows, category 1: LAD ends above the apex of the heart, category 2: LAD ends at the apex and category 3: LAD wrapping around the apex.

**Table 3:** Results of the adjusted logistic regression model in the subgroup analysis of patients with atherosclerotic coronary plaque rupture-related native coronary artery occlusion according to one-year mortality.

	Odds Ratio	95% CI	*p*
Thrombus mass	6.890	1.210–39.234	0.030
Diabetes mellitus	11.685	2.027–67.374	0.006
Ejection fraction	0.858	0.782–0.941	0.001
Neutrophil cell count	1.289	1.065–1.560	0.009

Adjusted for parameters, which showed a significant association with one-year mortality, as demonstrated in Table 2. Only parameters showing significant association with one-year mortality in this model and thrombus mass are demonstrated here.

## Data Availability

Data are available from the authors upon request.
